# The Importance of Arterial Blood Flow Detection for Risk Stratification and Eradication to Achieve Definitive Hemostasis of Severe Non-Variceal UGI Hemorrhage

**DOI:** 10.3390/jcm12206473

**Published:** 2023-10-11

**Authors:** Dennis M. Jensen

**Affiliations:** David Geffen School of Medicine at UCLA, Ronald Reagan UCLA Medical Center and The VA Greater Los Angeles Healthcare System, Los Angeles, CA 90095, USA; djensen@mednet.ucla.edu; Tel.: +310-268-3569

**Keywords:** arterial blood flow, doppler endoscopic probe, stigmata of recent hemorrhage, non-variceal UGI hemorrhage

## Abstract

Background: Non-variceal upper gastrointestinal bleeding (NVUGIB) is a common medical problem worldwide. Independent endoscopic risk factors for rebleeding and mortality of NVUGIB that are treatable are stigmata of recent hemorrhage (SRH) and arterial blood flow underneath SRH. The specific aims of this paper are to describe the importance of arterial blood flow detection for risk stratification and as a guide to definitive hemostasis of severe NVUGIB. Methods: This is a review of randomized controlled trials and prospective cohort study methodologies and results which utilized a Doppler endoscopic probe (DEP) for the detection of arterial blood underneath SRH, for risk stratification, and as a guide to definitive hemostasis. The results are compared to visually guided hemostasis based upon SRH. Results: Although SRH have been utilized to guide endoscopic hemostasis of NVUGIB for 50 years, when most visually guided treatments are applied to lesions with major SRH, arterial blood flow underneath SRH is not obliterated in 25–30% of patients and results in rebleeding. Definitive hemostasis, significantly lower rebleeding rates, and improvements in other clinical outcomes resulted when DEP was used for risk stratification and as a guide to obliteration of arterial blood flow underneath SRH. Conclusions: DEP-guided endoscopic hemostasis is a very effective and safe new method to improve patient outcomes for NVUGIB.

## 1. Introduction

**Figure 1:**
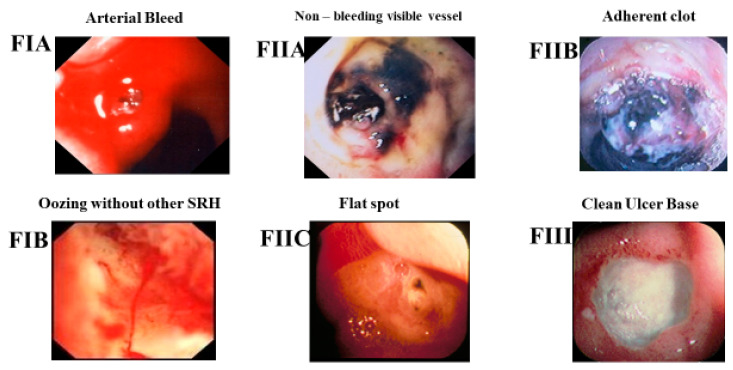
What are the Stigmata of Recent Ulcer Hemorrhage? Forrest—F—and Descriptive Classifications.

Ever since 1974, when Forrest first described stigmata of recent hemorrhage (SRH) as endoscopic risk factors for peptic ulcer bleeding (PUB), SRH seen on endoscopy (EGD) have been utilized by GI endoscopists worldwide as a visual guide for risk stratification of rebleeding and for endoscopic treatment and/or medical management of PUB patients hospitalized for severe upper gastrointestinal (UGI) hemorrhage [1]. Refer to Figure 1 for the Forrest classification of SRH for PUBs. The Forrest classification has been commonly used for the last 50 years by endoscopists in England, Europe, or Asia, whereas the corresponding descriptive classification is usually used in North America. Similar descriptive endoscopic SRH have been applied to other GI lesions with arteries beneath SRH to predict the potential risk of rebleeding in other non-variceal (NV) UGI and colon lesions including Dieulafoy’s lesions [2], Mallory Weiss (MW) tears [3], and colon diverticular hemorrhage [4].

For context on the application of SRH, my GI hemostasis research group approaches the diagnosis and endoscopic treatment based upon the type of GI lesion, its vascular anatomy, the type of underlying blood flow in the lesion (arterial, venous, or mixed), and whether the NVUGI lesion is focal or not. Refer to Table 1. Peptic ulcers, other UGI ulcers, Dieulafoy’s lesions, and MW tears have arteries underneath and focal bleeding sites. These NVUGI lesions with arterial blood flow underneath are the focus of this paper. In contrast, arteriovenous (AV) malformations are often multiple, are not bleeding at endoscopy, or have oozing bleeding including angiomas, gastric antral vascular ectasia (GAVE), telangiectasia, and portal hypertensive gastropathy. Other lesions with small vessels or capillaries, neovascularity, and diffuse oozing bleeding include UGI cancers, polyps, erosions, ischemia, and esophagitis. The latter two types of lesions with non-detectable AV blood flow by Doppler endoscopic probe (DEP) or with venous flow (e.g., varices or hemorrhoids) are not the focus of this paper, although they are often included in reports of different treatments for GI hemorrhage. 

In landmark histopathological studies of arteries in the base of PUB patients with either gastric (GUs) or duodenal ulcers (DUs), Storey, Swain, Johnston, and Chen reported that side holes in arteries underneath visible vessels (NBVVs) in peptic ulcers were the cause of both initial PUB bleeding and rebleeding [5,6,7,8,9]. Medical therapy with proton pump inhibitors (PPI’s)—in high intravenous (IV) infusions initially or later orally—can heal GUs and DUs and decrease the need for endoscopic therapy by reducing the prevalence of major SRH (e.g., according to Forrest, active arterial bleeding—FIA, NBVV—FIIA, or adherent clot—FIIB) on EGDs that are delayed [10]. However, medical therapy of PUBs without endoscopic hemostasis does not change the clinical outcomes of patients with severe UGI hemorrhage nor obliterate arterial blood flow underneath SRH in peptic ulcers [10]. In contrast, coaptive coagulation with thermal contact probes (multipolar—MPEC, bipolar electrocoagulation probes—BPEC, or heater probes) has been reported to be able to seal the walls together of arteries (up to about 2 mm in diameter) in laboratory studies and obliterate blood flow of arteries [11]. However, when applied clinically, these contact probes are not successful in obliterating arterial flow in 25–35% of peptic ulcers with major SRH, as documented by Doppler endoscopic probe (DEP) monitoring [12]. This high rate of residual blood flow after standard, visually guided endoscopic hemostasis (e.g., with thermal contact probes that can apply appositional pressure on the SRH or with through-the-scope hemoclips) is a major risk factor and predictor for PU rebleeding. This is the reason that such patients develop recurrent ulcer hemorrhage [12,13,14]. Patients with major SRH treated with visually guided standard endoscopic hemostasis rebleed about 25–30% of the time as reported in our recent prospective cohort studies and randomized controlled trials—RCTs [12,13,14]. Outcomes can be improved and providing recommendations about how to achieve this is one of the goals of this paper. 

Reports document the limitations of using Forrest SRH as the only endoscopic indicator of risk for more bleeding and as a guide to endoscopic hemostasis, including one from Lau et al. [15]. They reported that SRH as interpreted by international experts in a review of PUB SRH in coded endoscopic videos had low agreement rates in classifying different SRH and therefore, these SRH were not very accurate for the prediction of the risk of rebleeding [15]. This brought into question whether SRH alone are accurate for risk stratification and as a guide to endoscopic hemostasis. 

In an effort to improve risk stratification and GI hemostasis of NVUGIH, we and others have utilized DEP [2,12,13,14,16,17,18]. Based upon endoscopic SRH and underlying arterial blood flow monitoring with DEP, our group’s currently recommended goals of endoscopic hemostasis for severe, acute GI hemorrhage are (1) control of active bleeding and (2) prevention of rebleeding by obliteration of arterial blood flow underneath SRH in peptic ulcers and other GI lesions with underlying arteries [2,11,12,13,14,19,20]. 

### Overall Goals and Specific Aims

The overall goals of this report are to present recent advances in the endoscopic management of severe NVUGIH and to compare these with visually guided treatments based upon SRH and laboratory studies of hemostasis techniques and arterial blood flow. Also included are recommendations about ways to increase rates of definitive endoscopic hemostasis, decrease rates of rebleeding, and significantly improve clinical outcomes of patients with severe NVUGIH from peptic ulcers, Dieulafoy’s lesions, or MW tears. The specific aims of this report are as follows: (1) to describe the endoscopically identifiable risk factors that predict rebleeding of severe PUBs and NVUGIH, (2) to describe the DEP equipment and its utilization for detection of blood flow in GI focal lesions, (3) to detail the natural history of PUBs based upon detection of arterial blood flow underneath SRH, (4) to report results of DEP for risk stratification, prediction of rebleeding, and as a endoscopic guide for definitive GI hemostasis of focal NVUGI lesions with arterial blood flow detected underneath SRH, and (5) to report cost-effective studies of DEP as a management strategy for definitive GI hemostasis in comparison to traditional endoscopic visually guided treatments of SRH without DEP monitoring. 

## 2. Materials and Methods

Reported here are the CURE GI Hemostasis Research Group’s prospective cohort studies, natural history studies, and RCTs [2,12,13,14]. The results of RCTs and cohort studies by other investigators utilizing blood flow detection in NVUGIB will also be summarized and discussed [16,17,18,19,20]. The strengths, limitations, and clinical relevance of these reports will be discussed as they relate to current recommendations for endoscopic hemostasis and also the vascular anatomy type of GI lesion, SRH, and recommendations of a recent NIH consensus conference on studies of severe hemorrhage, including GI bleeding [21]. Other investigators have reported upon DEP monitoring of PUBs and describe their techniques and clinical results, both positive and negative [16,17,18,19,20]. Their methods and results will be reviewed and compared with ours. In a unique way, these studies illustrate the evolution, changes, and progress of GI endoscopic diagnosis, endoscopic risk assessment, and treatments that have been achieved and based upon these observations, what steps are recommended to optimize GI endoscopic hemostasis of NVUGIH and improve the clinical outcomes of patients. 

## 3. Results

The first CURE GI Hemostasis clinical trial illustrates the clinical outcome results and limitations of applying endoscopic hemostasis based upon visually SRH alone without monitoring blood flow underneath for patients with severe peptic ulcer bleeding (PUB) and major SRH—either spurting arterial bleeding (FIA) or NBVV (FIIA). This RCT was completed before DEP became available in the US. In that early RCT, patients were randomized to either medical–surgical treatment, BPEC, or heater probe endoscopic hemostasis [22]. All patients were treated with intravenous (IV) histamine 2 receptor antagonists and oral proton pump inhibitors (PPIs) because the IV PPIs were not yet available in the USA at that time. Unlike other countries such as those in Asia which have high prevalences of Helicobacter pylori (HP) infection as the etiology of PUs, in this study, about equal numbers of patients had aspirin or non-steroidal anti-inflammatory drug (NSAID)-induced or idiopathic peptic ulcers and HP-induced ulcers. HP-induced ulcers in PUB patients are known to be much more responsive to PPI treatment than HP negative ulcer patients in terms of reduced rebleeding rates, PU healing rates, and other outcomes [10,12,13,14,23,24]. In this CURE RCT, the managing physicians and other healthcare providers were blinded as to endoscopic treatment and made all subsequent management decisions about treatments, transfusions, and hospital discharge. Guidelines for endoscopic hemostasis that had been established with in vivo laboratory studies [11,25,26] were utilized for endoscopic treatments in patients. In this RCT, thermal contact probe coagulation with either heater probe or BICAP was applied in the ulcer base on the spurting bleeding point or NBVV with a large probe (e.g., 10 French size), firm tamponade, and low thermal energy in long treatment pulses (8–10 s) for each tamponade application [11]. The endoscopic treatment goals were to control active bleeding, flatten or cavitate the SRH, and achieve coaptive coagulation of the underlying artery [11]. However, during 30 days of prospective follow-up, the rebleeding rates for the medical–surgical, BPEC, and heater probe treatment groups were 66%, 40%, and 24%, respectively. In all-treatment groups, patients with continued bleeding or rebleeding had urgent surgery. The respective rates of urgent ulcer surgery in these treatment groups were 42%, 28%, and 5% [22]. Refer to Table 2 for other clinical outcomes from this RCT. Based upon observations in the laboratory about the importance of arterial blood flow in GI lesions [11], we postulated that the artery underlying the high risk SRH was not obliterated by the thermal coagulation and that continued arterial blood flow in the ulcer resulted in rebleeding [22]. 

Except for surgery and angiography, there were no other techniques available at the time to evaluate arterial blood flow underneath SRH before and after endoscopic treatment. Such a method to monitor arterial blood flow underneath SRH with a catheter applied endoscopically was needed to improve endoscopic risk stratification and guide endoscopic hemostasis. Different types of DEPs had been reported in early GI studies which were observational for most rather than interventional, the latter with the goal of obliterating blood flow [16,17,18,19,20]. In our subsequent investigations, DEP proved to be an invaluable tool for refining endoscopic risk stratification and as a guide to definitive endoscopic GI hemostasis for interventional studies. That is, by documenting the presence before and absence post-treatment of arterial blood flow underneath SRH in ulcers, Dieulafoy’s lesions, and MW tears in patients hospitalized with severe UGI hemorrhage [2,12,13,14,18,19]. These sequentially performed studies with DEP by the CURE GI Hemostasis Research Group are described herein. 

**Figure 2:**
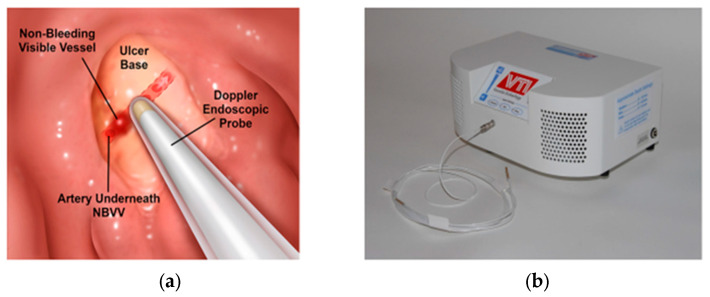
Detection of Arterial Blood Flow Underneath Stigmata of Recent Hemorrhage with Doppler Endoscopic Probe. (**a**) On the left is a diagram of an ulcer base with a non-bleeding visible vessel and the underlying artery tracked by a Doppler endoscopic probe. (**b**) On the right side is the control unit and a DEP catheter (Vascular Technology Inc., Nashua, NH, USA).

The second CURE Hemostasis report was of a prospective cohort study in patients with severe PUBs investigating whether DEP could improve the prediction of the risk of rebleeding when applied in the ulcer base to monitor arterial blood flow underneath SRH of different types before and after visually guided endoscopic hemostasis of PUBs [12]. The DEP technique and equipment are shown in Figure 2. Further technical details and results are presented in the publication [12]. Refer to Table 3 for a summary of study results of the 139 peptic ulcer patients with severe UGI hemorrhage who had DEP monitoring underneath SRH. The results are presented for each SRH including the detection rate of underlying arterial blood flow at baseline and after endoscopic visually guided hemostasis. That treatment was pre-injection of 1:20,000 epinephrine for active, spurting arterial bleeding (FIA) and adherent clots (FIIB—before clot removal with a rotatable snare), followed by application of either MPEC thermal coagulation and/or through-the-scope hemoclips (HCs) on the SRH.

All PUB patients were treated with IV PPIs in high doses except for oozing bleeding patients (FIB) who received PPIs orally, twice daily, in accordance with a recent report [27]. The overall detection by DEP of arterial blood flow under major SRH was 87.4%—with 100% in spurting arterial bleeders (FIA), 90.7% in NBVV (FIIA), and 68.4% with adherent clots (FIIB). The overall rate of detecting residual arterial blood flow of these major SRH after visually guided endoscopic hemostasis with DEP was 27.4%, with 35.7% in FIA (spurters), 27.4% in FIIA (NBVV), and 18.8% in FIIB (clot). For oozing bleeding (FIB) and flat spots (FIIC) in the ulcer base, the rates of arterial flow detection with DEP at baseline were 46.7% and 40.5%, respectively, while the corresponding residual blood flow rates after hemostasis were 0% for both these SRH. Based upon these prospectively collected results, my research group classified PUBs with oozing bleeding (F IB) or flat spots (F IIC) as “lesser SRH”. The combination of SRH and DEP monitoring of underlying arterial blood flow underneath SRH was used in subsequent interventional studies for patient management to stratify for the risk of rebleeding before and after visually guided endoscopic hemostasis. 

The third CURE Hemostasis Research Group’s RCT with DEP was an interventional study that enrolled 148 patients with severe NVUGIH [13]. In the DEP treatment group, blood flow underneath SRH was monitored and utilized for risk stratification and also as a guide to complete hemostasis, e.g., obliteration of arterial blood flow underneath SRH during the treatment. UGI lesions with SRH including ulcers (e.g., GUs, DUs, anastomotic ulcers, or esophageal ulcers with FIA, FIIA, and FIIB and lesser SRH—FIB (oozing) or FIIC (flat spot)—with arterial blood flow detected underneath); Dieulafoy’s lesions with active bleeding or NBVV; and MW tears with active bleeding. The distribution of lesions was 84.5% peptic ulcers, 12.8% Dieulafoy’s lesions, and 2.7% MW tears. The medical treatment of all PUB patients was high dose IV PPI infusions for 72 h followed by 30 days of BID PPIs [23]. Dieulafoy’s and MW tear patients received BID PPIs for 7 days and MW tear patients also received anti-emetics for the prevention of vomiting. The treatment groups were standard visually guided hemostasis (with MPEC or hemoclips with or without pre-injection of epinephrine as described above in the cohort study) or DEP for monitoring of arterial blood flow underneath SRH before and after DEP guided endoscopic hemostasis. If there was residual arterial blood flow after GI hemostasis in the DEP treatment group, either more MPEC treatment or more hemoclips could be applied if deemed to be effective and safe by the endoscopy investigator [13].

Refer to Table 4 for the clinical outcome results on rebleeding, surgery, complications, and death within 30 days. 

After the esophagogastroduodenoscopy (EGD), patients were managed by physicians and healthcare personnel who were blinded as to the endoscopic treatments. They made all post randomization management decisions about diagnosis and treatment for rebleeding (e.g., repeat endoscopic hemostasis, interventional radiology (IR) embolization, or surgery), transfusions, and discharge timing. This blinding of healthcare providers is a strength of our RCT and an important method to reduce potential bias. Similar blinding is lacking in other endoscopic RCTs and cohort studies with DEP that are reported [17,18,19,20].

The lesion rebleeding rate was significantly lower in the DEP group—11.1% (8 of 72)—than the standard treatment group—26.3% (20 of 76). Other clinically relevant outcomes were not significantly different [13]. In a sub-group analysis of the DEP treatment group, no patient (0 of 8) who had arterial blood flow obliterated with the endoscopic treatment had rebleeding. In contrast, the rebleeding rate of patients who had residual arterial blood flow after endoscopic hemostasis was 88.9% (8 of 9). This documents the high correlation and predictive value of DEP monitoring of arterial blood flow after visually guided endoscopic hemostasis and indicates that better visually guided treatments are needed which can safely and more effectively obliterate arterial blood underneath SRH, especially in large or fibrotic ulcers.

In an earlier RCT from Germany, Kohler et al. reported a study comparing endoscopic treatment based upon Forrest classification to DEP in endoscopic risk stratification and hemostasis of 100 patients with PUBs [19]. The patients had even distributions of risk by SRH including non-bleeding visible vessels (NBVV)—Forrest IIA (16 in Forrest vs. 18 in DEP); adherent clot—FIIB (10 vs. 10); flat spot—F IIC (10 vs. 8); clean ulcer base—FIII (14 vs. 14). Epinephrine injection was the treatment of NBVV and adherent clot cases for the Forrest group but only those in the DEP with arterial blood flow detected underneath the SRH. Overall, the initial rates of endoscopic treatment were 46% in the Forrest group and 52% in the DEP group. The clinical outcomes were significantly different as shown in Table 5.

The authors concluded that DEP monitoring of arterial blood flow underneath SRH of PUBs made definitive hemostasis possible by means of obliteration of the arterial blood flow which resulted in reductions in ulcer rebleeding, the need for emergency surgery, and all-cause mortality. This was a significant improvement over using SRH alone for definitive PUB hemostasis. This RCT did not include patients with spurting arterial bleeding (FIA). However, for the other major SRH (NBVV—FIIA and adherent clots—FIIB) as well as oozing bleeding (FIB), the results were clinically significant and important. As an evidence-based, well-designed RCT, their results are generalizable to PUB patients and other UGI non-variceal lesions with arterial flow underneath SRH. However, endoscopic treatments have improved with thermal coagulation, hemoclips, or combination hemostasis which obliterate underlying arterial blood flow more effectively than epinephrine injection treatment [2,11,12,13,14].

There are several previous cohort studies using DEP for risk stratification. Wong et al. reported a cohort study with DEP monitoring of PUB patients with SRH and visually guided endoscopic hemostasis [16]. Two separate endoscopy teams were involved; one performed the EGD and visually guided treatment of SRH and the other performed the DEP monitoring before and after the endoscopic treatment. The DEP findings were not disclosed to the team performing the endoscopic hemostasis. Therefore, this was an observational study and not an interventional study with DEP guiding hemostasis. Patients with a positive DEP signal for arterial blood flow underneath SRH after endoscopic hemostasis had a significantly higher recurrent PUB rebleeding rate than those with DEP negative signals (100% (3 of 3) versus 11% (1 of 9), *p* = 0.003) [16]. Their conclusion was that DEP can predict the failure of endoscopic therapy. 

In another cohort study, Jakobs et al. reported utilizing epinephrine injections for bleeding and non-bleeding SRH in 20 patients with PUBs [18]. They injected the epinephrine until the arterial signal disappeared and then followed up the patients to determine whether the epinephrine treatment resulted in definitive hemostasis. However, 20% (4 of 20) had recurrent ulcer bleeding and on second-look endoscopy, 27.7% were still DEP positive but did not rebleed. They concluded that after epinephrine injections, a negative DEP signal was not helpful clinically for confirming definitive hemostasis and no risk for rebleeding. In CURE studies, both in the laboratory and clinically with DEP, epinephrine injections have been observed to temporarily cause vasoconstriction with interruption of arterial flow, but this can be transient and the flow returns within an hour. More definitive modes of endoscopic hemostasis are now available and are recommended. 

In contrast to Jakobs et al.’s results with DEP monitoring of arterial blood flow underneath SRH in PUB patients, Kohler and Riemann reported that DEP monitoring, independent of Forrest classification, was clinically useful for risk stratification and as a guide to whether to use endoscopic treatment or not [17]. Sixty-six patients with DEP-positive ulcers were treated with epinephrine injections irrespective of their Forrest classification. Only 8% had rebleeding and none died of hemorrhage. None of the 40 DEP-negative PUB patients were treated endoscopically and none rebled, including 11 cases with Forrest IIA or IIB. Kohler et al. subsequently conducted an RCT of DEP as an interventional study which is discussed in detail above [19]. 

In another study from the Netherlands, van Leerdam et al. reported a multicenter study of PUB patients with non-bleeding SRH [20]. This report included both a randomized group of low-risk patients and a non-randomized higher risk group of patients monitored with DEP. They randomized patients with flat spots (FIIC) or clean ulcer bases(FIII) with positive DEP signals for arterial blood flow to medical therapy with PPIs vs. injection therapy with epinephrine. As expected for these PUB patients with low-risk stigmata, only one rebleed occurred (in the DEP-negative medical group). Also, there were no PUB surgeries, 30 day rebleeds, and no difference in RBC transfusions in these F IIC and F III low-risk patients. The authors used DEP to interrogate a separate group of PUB patients with oozing bleeding (FIB) or adherent clots (FIIB) and treated the 82% who had positive DEP with epinephrine endoscopic injection therapy. This group of higher risk patients were not randomized nor was an interventional trial using DEP to obliterate blood flow as an endpoint. However, with the 38 studied with DEP after epinephrine injection, the rebleeding rates of those patients with blood flow obliteration had a lower rebleeding rate than patients without obliteration (4% (1/27) vs. 27% (3/11), *p* = 0.06). 

There are several major study design limitations in this Dutch study which significantly reduce its generalizability and relevance to current clinical application [20]. First, the study was stopped before enrollment of the estimated sample size was reached. Second, the study did not include high-risk PUB patients with major SRH (FIA, FIB, FIIA, or FIIB) in the randomization. Third, only very low risk patients (FIIC and FIII) were included in the randomization, the numbers were small, and the differences in outcomes, as expected, were not different. Fourth, with a sub-optimally effective endoscopic epinephrine injection therapy in a study where obliteration of arterial blood flow was not the endoscopic hemostasis endpoint, no difference in rebleeding or other outcomes would have been expected. The authors concluded from their results that they failed to demonstrate a role for endoscopic DEP assessment in addition to SRH in assessing clinical decisions for PUB patients. Since this report, major improvements with thermal coagulation, hemoclipping, combination hemostasis (with thermal coagulation and/or hemoclips with or without epinephrine injection), and large over-the-scope clips have been introduced [2,12,13,14,22]. Also, a much larger, more rigorously designed RCT that includes both major and minor SRH has been reported by our group and is discussed above [13].

**Figure 3:**
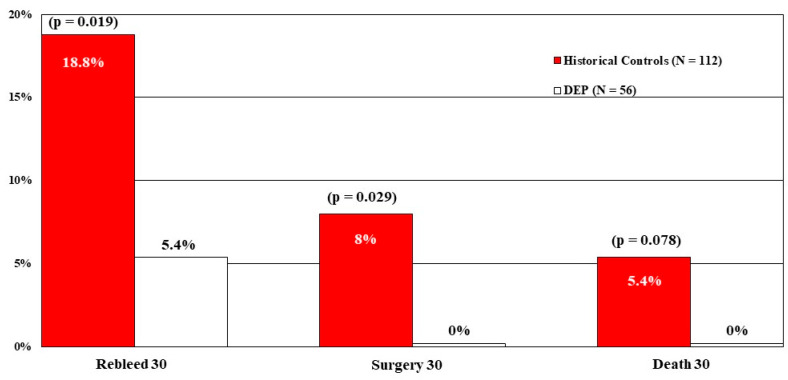
Comparison by Logistic Regression of RCT DEP PUB patients with matched historical controls. Historical controls from prior RCTs treated with visually guided hemostasis (*n* = 112) compared to DEP guided hemostasis (*n* = 56) with 30-day outcomes of rebleeding, surgery, or mortality.

Our fourth CURE study with DEP was a multivariate analysis (e.g., logistic regression) to compare the results of both DEP and standard treatment groups from the RCT [13] with carefully matched historical controls treated with visually guided endoscopic hemostasis in earlier CURE RCTs of patients with ulcers or Dieulafoy’s lesions [28]. The goal was to assess whether DEP was a major independent method of improving GI hemostasis over a longer time period for patients with PUBs and Dieulafoy’s lesions in terms of rebleeding, surgery, and mortality. Compared to 112 historical controls who had PUBs and visually guided treatments, 56 DEP-treated patients had significantly lower rates of PUB rebleeding and surgery, with mortality not significantly reduced, most likely due to the small number of deaths overall. See Figure 3.

**Figure 4:**
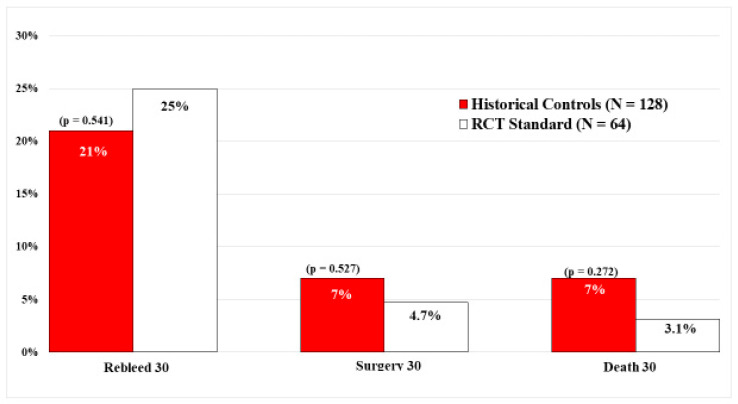
Comparison by Logistic Regression of RCT DEP PUB patients with matched historical controls. 30-day outcomes of historical controls of PUBs treated with standard endoscopic hemostasis (*n* = 128) compared to current standard endoscopic hemostasis (*n* = 64).

In contrast, there were no significant differences in the current standard visually guided treatment group of 64 PUB patients and 128 matched historical controls who also had visually guided endoscopic hemostasis. Refer to Figure 4. This is an important study that confirms and expands upon the results of the DEP RCT [13] by including rates of surgery and mortality in a much larger sample size of peptic ulcer patients. The study was based upon prior carefully performed sequential PUB RCTs by the same group of CURE investigators, in the same hospitals, and using standard visually guided endoscopic hemostasis techniques, based upon SRH, but at different times.

After the esophagogastroduodenoscopy (EGD), patients were managed by physicians and healthcare personnel who were blinded as to the endoscopic treatments. They made all post randomization management decisions about diagnosis and treatment for rebleeding (e.g., repeat endoscopic hemostasis, interventional radiology (IR) embolization, or surgery), transfusions, and discharge timing. 

In collaboration with the CURE Hemostasis Research Group and other investigators who used our results of the DEP RCT [13], two cost analyses studies were performed [29,30]. DEP was reported to be significantly more cost effective than standard visually guided GI hemostasis in the first study for PUB patients with all types of SRH [29]. In another cost effectiveness study utilizing our DEP RCT results with flat spots (F IIC) in severe PUBs, DEP was also reported to be a more cost-effective treatment than medical treatment alone [30].

**Figure 5:**
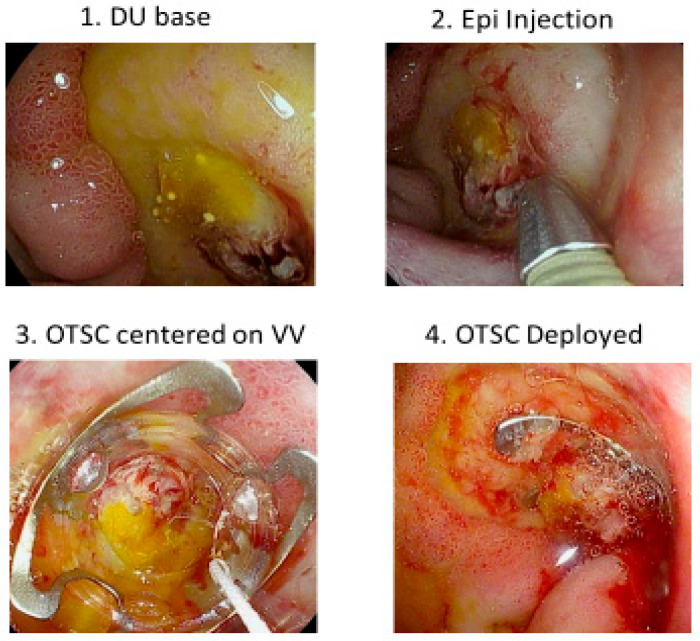
OTSC Hemostasis of a Giant DU with Bleeding Visible Vessel. (**1**) Giant duodenal ulcer with a Visible Vessel (VV) in the base (**2**) Pre-injection of 1:20,000 epinephrine around the VV (**3**) Centering OTSC on the VV (**4**) After OTSC deployment on the VV in the ulcer base.

Our next CURE Hemostasis study was an observational study to evaluate the ability of large over-the-scope-clips (OTSCs) to obliterate arterial blood flow underneath SRH in peptic ulcers and to prevent rebleeding in patients with severe UGIH within 30 days of follow-up [31]. Refer to Figure 5 as an example of OTSC hemostasis of a giant DU with bleeding from a visible vessel. Results were compared with those of other patients treated with standard visually guided hemostasis based upon SRH (e.g., Forrest classification) or DEP guided hemostasis. Whereas residual blood flow and rebleeding were seen in 26–27% of PUB patients with visually guided hemostasis, with OTSC both those rates were only 4%, and for DEP guided endoscopic treatment with eradication of arterial blood no patient had peptic ulcer rebleeding [31]. 

Another CURE GI Hemostasis Research Group RCT compared the outcomes of OTSC as initial treatment with standard visually guided endoscopic hemostasis for patients with peptic ulcer or Dieulafoy lesions and severe UGIH [14]. Similar to the DEP RCT, patients were managed by physicians and healthcare personnel who were blinded as to the endoscopic treatments. They made all post randomization decisions about the diagnosis and treatment for rebleeding (e.g., repeat endoscopic hemostasis, IR embolization, or surgery), transfusions, and discharge timing. Overall, patients treated with OTSC had a significantly lower rebleeding rate than the standard endoscopic treatment group (4% vs. 28.6%). However, these rates were only significantly different for patients with major SRH: spurting arterial bleeding (FIA), NBVV (FIIA), and adherent clot (FIIB). The RCT patients with major SRH accounted for all the rebleeding, with 5.9% (1 of 17 patients) in the OTSC group versus 34.8% (8 of 23 patients) in the standard endoscopic treatment group. No patient with lesser SRH (e.g., oozing bleeding (FIB) or flat spots (FIIC)) had rebleeding [14]. Therefore, we concluded that not all patients with different SRH in ulcers can be expected to benefit from OTSC. Furthermore, there are other limitations of OTSCs and their deployment. The OTSCs are more expensive than standard hemoclips, MPEC, or DEPs, additional expertise is needed to successfully deploy OTSC onto the SRH of lesions with severe NVUGIH, and there is a moderate learning curve which requires the performance of several emergency treatments to be competent [14,32,33]. Accurate deployment of OTSCs onto the SRH for definitive hemostasis of fundal gastric lesions in retroflexion, angulated anastomotic ulcers, and duodenal bulbar ulcers can also be especially challenging [14,33]. 

The last CURE study is a cohort study comparing rebleeding rates of 82 consecutive patients with Dieulafoy’s lesions treated by the CURE GI Hemostasis Research Group using different types of endoscopic methods [2]. In that report, patients treated with DEP-guided hemostasis to obliterate underlying arterial blood flow had the lowest rebleeding rates when compared to visually guided standard treatments without DEP monitoring. Using propensity score matching of Dieulafoy’s lesion patients based upon clinical and endoscopic risk factors, the 30-day rebleeding rate of the visually guided treatment group was significantly higher than the DEP treatment group—25.3% versus 2.6%, respectively, (*p* < 0.001). For a composite outcome of rebleeding, surgery, angiography, severe complications, and death [21], the adjusted propensity score was also significantly worse for the visually guided treatment group than the DEP group—39% vs. 2.6% [2]. 

## 4. Discussion

Conclusions based upon our CURE Hemostasis Research studies and those of others reported are as follows: (1) The type of vessels (e.g., artery, vein, AV malformation, or neovascularity) and the type of blood flow underneath non-variceal UGI lesions that cause acute hemorrhage are a major determinant of the severity of GI bleeding and the risk of rebleeding. Refer to Figure 1 and Table 1. (2) The Forrest classification alone is not an accurate nor reproducible guide to risk stratification, the treatment of peptic ulcers, or other NVUGI lesions with arteries underneath SRH [12,27]. (3) Forrest IA (spurting arterial bleeding) lesions have significantly higher rebleeding rates after endoscopic treatments than Forrest IB (oozing bleeding). F IA and F IB patients should not be combined as “active bleeders” and reported together in GI outcome studies because their natural history is significantly different [12,27]. (4) DEP monitoring of arterial blood flow underneath SRH in peptic ulcers, Dieulafoy’s lesions, and Mallory Weiss tears is a major improvement over utilizing SRH alone for risk stratification and as a guide to endoscopic hemostasis of these lesions [12,13]. (5) When used in interventional GI hemostasis studies, DEP monitoring of arterial blood flow underneath SRH in peptic ulcers, Dieulafoy’s lesions, and MW tears for risk stratification and as a guide to GI hemostasis significantly reduces rebleeding rates compared to visually guided treatment based upon lesion SRH alone [13,28]. (6) This new treatment is both safe and cost effective [13,29,30]. (7). Whereas large OTSCs are more effective than visually guided endoscopic treatment with through-the-scope hemoclips or MPEC hemostasis in recent RCTs, only those patients with major SRH (spurting arterial bleeding (Forrest IA), NBVV (FIIA), or adherent clot (FIIB)) significantly benefited from this new type of endoscopic treatment. Patients with lesser SRH—oozing bleeding (FIB) or flat spots (FIIC) with underlying blood flow—had very low rebleeding rates after standard visually guided endoscopic hemostasis and did not benefit from the more expensive and technically more difficult treatment with large OTSCs [14]. 

It is of interest to update results of PUB patient’s prognosis and natural history of hemorrhage for oozing (FIB) PUBs and spurting (FIA). Recent RCTs with OTSC in comparison with standard visually guided treatment confirm what has been previously reported [12,13,14]. Lau et al. recently reported a large RCT of OTSC vs. standard GI hemostasis for patients hospitalized for NVUGIH in Asia [33]. A summary of combining the results for PUBs of our OTSC RCT [14] and the Lau RCT [33] according to major and lesser SRH is shown in Table 6. For OTSC vs. standard GI hemostasis of severe PUBs, 163 patients with peptic ulcer hemorrhage and major SRH had a significant difference in rebleeding rates. These respective rates of PUB rebleeding were 4.7% vs. 26.7% (with *p* value of 0.0005, 95% confidence intervals (CIs) of 8.2% and 31.1%, and the number needed to treat (NNT) of 5. In contrast, for the lesser SRH of oozing bleeding (Forrest IB) and flat spots (FIIC) in 65 PUB patients, the rebleeding rates for OTSC and standard GI hemostasis were 0% and 5.7%, respectively. These latter differences were not significant with a *p* value of 0.4952 (95% CI of –5.9% to 19.2%). 

Both Lau and our group described these as potential limitations of using OTSC in all NVUGI lesions and both commented about other technical difficulties and challenges of OTSC compared with standard through-the-scope hemostasis techniques [14,33]. These include stenosis or strictures of the esophagus or pylorus (so that the large OTSCs cannot be passed), difficulty of deployment in the fundus or in angulated positions such as the duodenal bulb or surgical anastomoses, and the extra training and experience needed for success with OTSC. Also, for OTSC treatments, the endoscope must be withdrawn after initial diagnosis and preparation of the bleeding lesion (with suctioning of blood and clots and pre-injection of epinephrine if needed). Then re-insertion of an endoscope with the OTSC and deployment on the SRH can be performed. Removal and re-intubation of the patient with the OTSC on an endoscope takes extra time compared to utilizing the same endoscope for both diagnosis and treatment in standard hemostasis procedures. For recurrent bleeding from peptic ulcers, Schmidt et al. also reported in a multicenter RCT that OTSC treatment was more effective than standard endoscopic treatment, with hemoclipping usded in most patients [34]. In my GI Hemostasis group, DEP-guided monitoring for risk stratification combined with OTSC or combination treatment with MPEC and HCs is currently recommended to achieve definitive hemostasis of peptic ulcers, Dieulafoy’s lesions, or MW tears which rebleed after standard endoscopic treatment, angiography, or surgery.

A summary of DEP-guided treatment, OTSC, and standard endoscopic hemostasis techniques; recommended training; and clinical results about lesion rebleeding is presented in Table 7. Whereas initial GI hemostasis rates of over 95% are reported with each of these techniques, the actual lesion rebleeding rates are significantly higher with standard endoscopic hemostasis in patients with severe NVUGIH and major SRH [12,13,14,31]. However, the definitive hemostasis rates for lesser stigmata (FIB (oozing) and FIIC (flat spots) with positive underlying arterial blood flow) are very high. Limitations of OTSC are lesion access for accurate deployment, strictures or stenosis, additional cost, and that more training is required to achieve successful deployment onto the SRH. 

As an additional overview about blood flow monitoring in GI lesions that bleed, different techniques for the treatment of GI hemorrhage have current potential indications; most do not obliterate the blood flow underneath the GI lesion and therefore, they are not definitive but can be palliative. Topical agents such as hemospray and clotting factors are not capable of obliterating blood flow underneath lesions as they slough off within 1–2 days, and therefore, the lesion is exposed with its underlying blood flow anatomically unaltered. Topical agents may have a palliative role in temporarily stopping or slowing GI bleeding from diffuse mucosal lesions, ischemia, erosions, neoplasms, or other lesions. However, definite hemostasis will be required later to prevent rebleeding from focal lesions such as peptic ulcers, Dieulafoy’s lesions, or MW tears with major SRH and arterial blood flow from underneath. Heater probe for thermal coagulation is no longer commercially available since this effective device was withdrawn from the market by Olympus Corporation. Argon plasma coagulation (APC) and lasers are non-contact coagulation methods which are only capable of coagulation of small arteries less than 0.25 mm [11] and are not effective in obliterating arterial blood flow in focal lesions with major SRH that have larger arteries [5,6,7,8,9]. For oozing bleeding (F IB), APC or lasers may be effective in coagulating small underlying vessels but not large ones without eroding into them [11]. Bipolar or MPEC probes are readily available, inexpensive (compared to OTSC), and can obliterate blood flow underneath major SRH up to about 75% of ulcers, Dieulafoy’s lesions, or MW tears cases when visually directed on the SRH with the goal of coaptive coagulation [11,12,13,14]. However, rates of lesion rebleeding can be dramatically improved if arterial blood flow underneath SRH is monitored with DEP and further endoscopic hemostasis is applied to eradicate the blood flow [14,28]. When this is the endpoint of endoscopic treatment (such as with combination MPEC and HCs or OTSC), no lesion rebleeding has been reported [14,28]. The advantages of standard through-the-scope GI hemostasis with MPEC and HCs in combination with DEP monitoring are that it is faster, as effective, and safer than OTSC. As a reported example of complications, OTSC can cause stenosis of the lumen and obstruction if applied in a narrow lumen [33]. Through-the-scope hemostasis treatments do not have the same limitations of OTSC in terms of strictures (smaller endoscopes can be used and dilatation can be performed), training (MPEC and HCs are easy to use; DEP is easy to learn), and expense [14,32].

What will be required to improve GI endoscopic hemostasis of severe NVUGIH in clinical practice? There needs to be a concerted effort by GI training program directors, GI endoscopists, and GI endoscopic societies. The training about NVUGIH should be improved in several ways [32]. These include incorporating new cognitive and technical training about the vascular anatomy of different GI lesions that cause severe GIH; teaching about and utilizing DEP monitoring along with visual SRH to refine and improve endoscopic risk stratification into major and lesser SRH of focal NVUGI lesions; and utilizing DEP-guided hemostasis, OTSC, and other new effective methods for definitive hemostasis of patients with severe NVUGIH with major SRH and also other patients with severe rebleeding. Standard visually guided treatments are effective for lesser SRH (e.g., oozing bleeding (Forrest IB) or flat spots (FIIC) and endoscopists should be encouraged to utilize these. This training will depend upon a renewed emphasis by GI societies on GI hemorrhage, updating guidelines for clinicians on vascular anatomy and risk stratification, and most of all, on skilled bedside training by experienced endoscopists and teachers who want to improve patient outcomes for all types of GI hemorrhage, including NVUGIH [32]. 

## Figures and Tables

**Table 1:** Grouping NVUGI lesions by vascular anatomy, type of blood flow in the lesion, and whether the lesion is focal or not.

• Arteries beneath/focal bleed sites: PUBs, other GI Ulcers (PPIU’s & EMR’s); Dieulafoy’s lesions; MWT tears.
• AV malformations/multiple/diffuse bleeding: angiomas, GAVE, radiation telangiectasia, and portal hypertensive gastropathy.
• Small vessels or neovascularity/diffuse bleeding: UGI cancers, polyps, erosions, esophagitis, Cameron ulcers, and ischemia.

**Table 2:** Comparison of Bipolar Coagulation, Heater Probe, and Medical–Surgical Treatment for Ulcers with Spurting Arterial Bleeding or Non-bleeding Visible Vessels in a CURE RCT.

	Medical (*n* = 41)	Bipolar (*n* = 45)	Heater Probe (*n* = 41)
Initial hemostasis	14%	93% *	95% *
Further bleeding	66%	40%	24% *
Surgery for bleeding	42%	28%	5% *
Blood transfusion (units)	3.4	2.4	1.0 *
Hospital days (mean)	11.3	10.8	7.7
Mortality	10%	2.3%	2.4%

* *p* < 0.05.

**Table 3:**
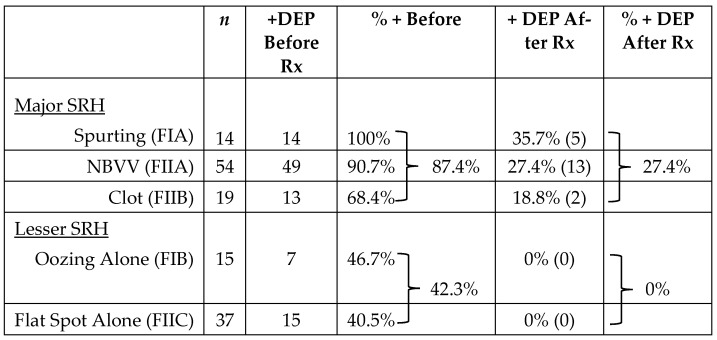
Stratification of PUBs by SRH and Reporting of Arterial Blood Flow Underneath Before and After Standard Endoscopic Hemostasis.

**Table 4:** 30 day Outcomes in an RCT of Doppler Probe vs. Standard Hemostasis for Severe NVUGIB.

	* Standard	Doppler	*p* -Value
Patients	76	72	
Same rebleed	20 (26.3%)	8 (11.1%)	0.021
Surgery for rebleed Angio for rebleed	4 (5.3%) 1 (1.3%)	0 (0%) 4 (5.6%)	0.120 0.200
Death	3 (4.0%)	1 (1.4%)	0.337
Major complications	4 (5.3%)	0 (0%)	0.120
Perforations	1 (1.3%)	0 (0%)	
CVA	2 (26%)	0 (0%)	
Pneumoperitoneum	1 (1.3%)	0 (0%)	
Other GI bleeds	3 (4.0%)	4 (5.6%)	0.714

148 RCT patients with peptic ulcers—84.5%, Dieulafoy’s lesions—12.8%, and MWT—2.7%. * Standard Hemostasis was MPEC and/or hemoclips with or without pre-injection of epinephrine.

**Table 5:** Kohler et al. RCT of PUB vs. DEP Treatment Based Upon Forrest Classification [19].

	Forrest Group *n* = 50	Doppler Group *n* = 50	*p* Value
Epinephrine Injection Therapy	46% (23)	52% (26)	
Rebleeds	14% (7)	2% (1)	<0.03
Emergency surgery	10% (5)	0% (0)	0.02
Bleeding-related death	4% (2)	0% (0)	0.15
All-cause mortality	10% (5)	0% (0)	0.02

**Table 6:** Which Patients with Severe UGI Bleeding from PUBs Benefit from Initial OTSC Treatment and Who Does not?

Treatments	OTSC	STANDARD
**Rebleeding Rates—Major SRH**		
FIA (Spurt)	1/12	5/12
FIIA (NBVV)	3/70	12/62
FIIB (Clot)	0/3	2/4
**Subtotal Major SRH**	**4/85 (4.7%)**	**21/78 (26.9%)**
**Treatments**	**OTSC**	**STANDARD**
**Rebleeding Rates—Lesser SRH**		
FIB (Ooze)	0/24	2/32
FIIC (Spot with + DEP)	0/6	0/3
**Subtotal Minor SRH**	**0/30 (0%)**	**2/35 (5.7%)**

228 patients (222 peptic ulcer and 5 Dieulafoy’s lesions). Results of CURE (Jensen Ref. [14]) and Asian Pacific (Lau Ref. [33]) RCTs. For major SRH, the *p*-value is 0.0005, 95% CIs (8.2% and 31.1%), and NNT of 5. For lesser SRH, the *p*-value is 0.4952 and 95% CIs (−5.9% and 19.2%).

**Table 7:** Comparison of OTSC, Standard Visually Guided Hemostasis, and Doppler-Guided Hemostasis Treatments for Initial Hemostasis of Severe NVUGIH.

	OTSC	STANDARD VISUALLY GUIDED HEMOSTASIS	DOPPLER-GUIDED HEMOSTASIS
Additional Training Required	Yes	Yes	Yes
ENDPOINT: Control Arterial Bleeding Success Rate	>95%	>95%	>95%
Obliteration Rate of Arterial Blood Flow- Major SRH	>90%	75%	>95%
Definitive Hemostasis Rate Major SRH (No Crossover)	>90%	75%	>95%
Definitive Hemostasis Rate Lesser SRH	95%	95%	100%
Potential Limitations of Deployment	Access, Strictures, and Stenosis	None	None
EGD Time for Diagnosis and Hemostasis	Longer	Shorter	Shorter
Cost of Hemostasis Devices	More	Less	Less

## Data Availability

De-identified data presented in these studies are available on request from the corresponding author.
